# Does Anemia Have a Potential Effect on Type 2 Hepatorenal Syndrome?

**DOI:** 10.1155/2020/1134744

**Published:** 2020-12-17

**Authors:** Sondes Bizid, Haythem Yacoub, Ghanem Mohamed, Bahaa Ben Slimane, Khouloud Boughoula, Hatem Ben Abdallah, Riadh Bouali, Nabil Abedelli

**Affiliations:** ^1^Gastroenterology and Hepatology Department, Military Hospital of Tunis, Tunis, Tunisia; ^2^Faculty of Medicine of Tunis, El Manar University, Tunis, Tunisia

## Abstract

*Background*/*Aims*. Hepatorenal syndrome (HRS) is a form of functional renal failure arising in advanced cirrhosis and is characterized by a poor survival rate. Anemia is frequently observed during the clinical course of cirrhosis. Our study aimed to investigate the hematologic findings in patients with cirrhosis to determine the effects of anemia on renal functions in type 2 HRS and if it was a potential aggravating factor. *Materials and Methods*. This prospective study, in which all consecutive patients with cirrhosis were enrolled, was performed at a tertiary-level hospital (Military Hospital of Tunis) from January 2019 to June 2019. A total of 9 patients with HRS fulfilled the type 2 HRS diagnostic criteria, and 41 patients with cirrhosis without HRS were included. All data regarding patients were obtained from the medical record. Demographic data, routine hemograms, biochemical, and urinary test results were collected. Models of end-stage liver disease (MELD) and Child–Turcotte–Pugh (CTP) scores were calculated. *Results*. The most common etiology of cirrhosis was viral hepatitis (66%). According to the CTP score, 23 patients were in the CTP-A stage, 13 in the CTP-B stage, and 14 patients were in the CTP-C stage. Patients with type 2 HRS had significantly lower hemoglobin levels compared with non-HRS stable cirrhosis patients. As hemoglobin levels decreased, renal function worsened on patients with type 2 HRS. Patients with lower hemoglobin levels had poor prognosis and survival compared with patients with higher hemoglobin levels. Logistic regression analysis showed that lower hemoglobin levels and higher MELD and CTP scores were statistically significant for an onset of type 2 HRS. *Conclusion*. Renal dysfunction is a frequent complication in patients with end-stage chronic liver disease. The role of anemia in aggravating HRS in patients with cirrhosis is explained by hypoxia that can lead to microcirculatory renal ischemia. Other studies are required to determine if anemia is a precipitant factor for HRS or not.

## 1. Introduction

Hepatorenal syndrome is a form of functional renal failure arising from liver cirrhosis secondary to diminished renal blood flow [1]. Typically kidneys are histologically normal [2]. Portal hypertension leads to vasodilatation of splanchnic vessels, decrease in systemic vascular resistance, and activation of the renin-angiotensin-aldosterone causing vasoconstriction of renal arteries [3]. Characteristically, HRS affects cirrhotic patients with ascite. Two types of HRS have been described. Type 1 is characterized by acute rapid decrease in kidney function and progressive kidney failure in less than 2 weeks. Prognosis is poor with high mortality (90%) within 3 months. Type 2 HRS presents as a less severe kidney failure and gradual decline in renal function and longer survival than type 1 HRS [4]. The rate of progression of renal impairment is the main diagnosis criteria between the two types of HRS.

Anemia causes microcirculatory tissue hypoxia. This phenomenon is called anemic hypoxia. Renal ischemia is the most common cause of HRS. Renal tissue hypoxia or ischemia can trigger the initial tubular damage [5].

Anemia is frequently observed during the clinical course of cirrhosis. There are rare publications addressing the potential or additive effects of anemia on HRS in cirrhotic patients.

Our study aimed to investigate the hematologic findings in patients with cirrhosis to determine the effects of anemia on renal functions in type 2 HRS and if anemia was a potential aggravating factor.

## 2. Materials and Methods

### 2.1. Study Design

A prospective study in which all consecutive patients with cirrhosis were enrolled was performed at a tertiary-level hospital (Military Hospital of Tunis) from January 2019 to June 2019. This study was performed according to the Declaration of Helsinki, following the guidelines for good clinical practice, and the ethics committee approved the study protocol.

The diagnosis of cirrhosis was based on the clinical and laboratory signs of hepatocellular failure and portal hypertension such as stellar angioma, hepatic encephalopathy, low serum albumin level, prolonged prothrombin time, ascites, jaundice, esophageal or gastric varices, and irregular hepatic echogenicity of the liver in abdominal US.

Etiologies of cirrhosis were various including viral hepatitis, autoimmune hepatitis, primary biliary cholangitis, nonalcoholic steatohepatitis, and unknown etiologies.

All data regarding patients were obtained from the medical record. Demographic data (sex, age), routine hemograms, biochemical, and urinary test results were collected. Models of end-stage liver disease (MELD) and Child–Turcotte–Pugh (CTP) scores were calculated.

Postrenal dysfunction causes were excluded. The exclusion criteria were determined as shock, current, or recent treatment with nephrotoxic drugs, proteinuria >500 mg/day, infection, urinary red blood cells >50 per high-power field (HPF), abnormal findings on renal ultrasonography, and patients without complete HRS diagnostic criteria. Acute kidney injury is defined as a serum creatinine increase of ≥0.3 mg/dL (26.5 *μ*mol/L) within 48 h or of ≥50% from baseline within 7 days. No improvement of serum creatinine after two days of diuretics withdrawal and volume expansion with albumin (1 g/kg of bodyweight per day up to a maximum of 100 g/day) is required to exclude prerenal acute kidney injury (AKI) due to volume depletion and retain the diagnosis of HRS on patients with AKI.

Patients whose hemoglobin levels were less than 13 g/dl in males and 12 g/dl in females were considered with anemia. Hypersplenism was diagnosed in patients with pancytopenia, splenomegaly on abdominal US, and normal laboratory findings (haptoglobin, serum vitamin B12 and folate level, serum ferritin, and the direct Coombs test). Low serum levels of ferritin led to the diagnosis of iron deficiency anemia. Immune hemolytic anemia was diagnosed by the increased serum lactate dehydrogenase level and direct Coombs positivity. Vitamin B12 and folate deficiencies were the diagnoses when the serum levels were lesser than 150 pg/ml and 5 ng/ml, respectively. All patients were followed in-hospital and out-of-hospital for at least six months.

### 2.2. Patients Groups

Patients were divided into two groups:

- Type 2 HRS group: these patients had a serum creatinine increase of ≥0.3 mg/dL (26.5 *μ*mol/L) within 48 h or of ≥50% from baseline within 7 days
- Non-HRS group: patient with cirrhosis and without renal dysfunction

### 2.3. Statistical Analysis

The data were analysed on the Statistical Package for the Social Sciences (SPSS) version 21, IBM SPSS Inc., Chicago, IL, USA). Results were expressed as mean ± standard deviation. Univariate comparisons between groups were performed using chi-squared tests for dichotomous variables. For continuous variables, independent samples *t*-tests were used. A binary logistic regression model was used for introduction to HRS. A spearman's bivariate correlation was used to correlate serum creatinine values with the MELD score, CTP score, hemoglobin, ascite, and albumin. A *p* value of <0.05 was considered to be significant.

## 3. Results

Fifty patients (9 with type 2 HRS; 41 without HRS) with cirrhosis were included in our study. Only 9 patients fulfilled the criteria of type 2 HRS. Of these 50 patients, 27 (54%) were male and 23 (46%) were female. The mean age of our patients was 62.8 ± 11.9 years (range 14–86), and there was no statistically significant difference between the age of the two groups (*p*=0.129).

According to the CTP score, 23 patients (46%) were categorized in Grade A, 13 (26%) were in Grade B, and 14 (28%) in Grade C, respectively. Of the total of 9 patients with HRS, 3 (33.3%) were in the CTP-B stage, and 6 (66.7%) were in the CTP-C stage (Table 1). The majority of non-HRS patients were in the CTP-A stage.

Etiologies of liver disease in patients included in this study were hepatitis B in 20 patients (40%), hepatitis C in 11 patients (22%), nonalcoholic steatohepatitis in 5 patients (10%), primary biliary cirrhosis in 1 patient (2%), sarcoidosis in 1 patient (2%), autoimmune hepatitis in 1 patient (2%), primary sclerosing cholangitis in 1 patient (2%), and finally, cryptogenic cirrhosis in 10 patients (20%).

In this study group, the mean hemoglobin rate was of 10.24 ± 2.58 gr/dl (Table 2). The mean hemoglobin of HRS type 2 and non-HRS was 7.62 ± 2.45 g/dl and 10.82 ± 2.25 g/dl, respectively. Anemia was observed in 72% of patients. There was a statistically significant difference between the mean hemoglobin of the two groups (*p* < 0.001). The mean MCV of the whole study group was 85.76 ± 9.8 fl.

Hypersplenism was the most common cause of anemia among the various causes, representing 50% of cases followed by iron deficiency anemia in 36.1% and immune hemolytic anemia in 13.9% of cases (Table 2).

There were no statistical differences between the groups in terms of age, gender, and platelet count. The demographic features and laboratory data of the study groups are shown in Table 3.

In patients with type 2 HRS, there was a correlation between serum creatinine and lower hemoglobin and albumin levels (*r* = −0.333, *p* = 0.018; *r* = −0.403, *p* = 0.04, respectively), higher MELD and CTP scores (*r* = 0.618, *p* < 0.001; *r* = 0.796, *p* < 0.001, respectively), and the presence of ascite (*r* = 0.410, *p* = 0.03), which are indicators of the degree of liver failure. As hemoglobin levels decreased, renal function worsened attested by the increased serum creatinine level in the patients with type 2 HRS. There were no significant correlations between serum creatinine levels, bilirubin level hematocrit, and the presence or not of encephalopathy.

Hemoglobin levels, CTP score, and MELD score were included in logistic regression analysis. Hemoglobin and MELD score were statistically significant (*p* = 0.049 and *p* = 0.021, respectively) (Table 4).

Six patients (66.66%) died; three died during hospitalization, while 3 died out-of-hospital. The overall survival was 5.2 months. The mortality was higher in patients with HRS compared with those with no HRS (*p* < 0.001). Lower hemoglobin levels showed a significant association with mortality in all patients with HRS type 2 (*p* = 0.038). CTP and MELD scores were higher in nonsurvivors (Table 5).

## 4. Discussion

In our study, we prospectively reviewed the presence and the absence of type 2 HRS and anemia in 50 chronic liver disease patients. Etiology and morphology of anemia were reported. The diagnosis of cirrhosis was based on the clinical and laboratory signs of hepatocellular failure and portal hypertension such as stellar angioma, encephalopathy, low serum albumin level, prolonged prothrombin time, ascites, jaundice, esophageal or gastric varices, and irregular hepatic echogenicity of the liver in abdominal US.

Etiologies were various including viral hepatitis (66%), autoimmune hepatitis (2%), primary biliary cholangitis (2%), primary sclerosing cholangitis (2%), sarcoidosis (2%), nonalcoholic steatohepatitis (10%), and unknown etiologies (20%).

According to the CTP score, 23 patients were in the CTP-A stage, 13 in the CTP-B stage, and 14 patients were in the CTP-C stage. Anemia was observed in 72% of patients, comparable to literature [6].

Patients with type 2 hepatorenal syndrome had significantly lower hemoglobin levels compared with non-HRS stable cirrhosis patients. As hemoglobin levels decreased, renal function worsened attested by an increased serum creatinine level in the patients with type 2 HRS. Patients with lower hemoglobin levels had poor prognosis and survival compared with patients with higher hemoglobin levels.

Despite the fact that HRS is a functional syndrome, it is still associated with a high mortality and poor prognosis [7]. Renal injury in HRS is defined as a decrease in urinary sodium excretion and urine output. Proteinuria is absent, and the kidney histological structure is normal without evidence of pathology [8].

In normal conditions, the liver plays a significant role in the regulation of renal function. Hepatic regulation of renal function involves a hepatorenal reflex which is elicited by an increase in intrahepatic pressure and amino acids in portal venous blood. This reflex leads to a marked decrease in renal blood flow and glomerular filtration [9]. Portal hypertension leads to vasodilatation of splanchnic vessels, decrease in systemic vascular resistance, and activation of the renin-angiotensin-aldosterone causing vasoconstriction of renal arteries, decreased renal perfusion, and renal failure [3]. Splanchnic dilation seems to result from increased production of nitric oxide (NO). NO is excessively produced in cirrhosis as a result of increased shear stress on splanchnic and systemic circulation [10]. NO increases renal vasoconstriction, which contributes to the observed decline in renal function in HRS [11, 12]. Precipitating factors of HRS are various including spontaneous bacterial peritonitis, variceal bleeding, large volume paracentesis, and over use of diuretics [13, 14].

Anemia was more frequent with advanced liver disease. Hemoglobin levels dropped when the MELD score increased [15]. The frequent occurrence of anemia in patients with cirrhosis is explained in certain degree by the role of hepatocyte on red blood cell production and the enterohepatic cycle in supplying the vitamins necessary for hematopoiesis [16]. Many other contributing factors may lead to anemia: hypersplenism, inflammatory conditions, iron deficiency, malnutrition, bone marrow depression, and gastrointestinal hemorrhage. Many patients manifest more than one of them. A Spanish study showed that low hemoglobin concentration worsens the hyperdynamic circulation parameters in patients with portal hypertension. Anemia may accelerate the development of gastrointestinal bleeding, ascites, and hepatorenal syndrome, known to be as complications related to portal hypertension [17]. Therefore, anemia reduces oxygen supply to the kidneys and may contribute to microcirculatory renal hypoxia and injury. Consequently, renal tissue hypoxia promotes the initial tubular damage, leading to AKI [18]. Anemia also aggravates hyperdynamic circulation. In patients with cirrhosis, anemia increases hospital mortality rate [19].

A strong positive correlation was observed between anemia and the risk of poor outcomes in patients with end-stage renal disease [20].

In cardiology, anemia and iron deficiency are often associated with less good prognosis on patients with heart failure. Correction of the anemia improves functional capacity and decreases hospitalizations, but does not ameliorate mortality in these patients [21]. Another study showed that anemia is an independent predictor of death and major clinical adverse events in elderly patients diagnosed with stable coronary artery disease [22, 23].

Studies have shown a strong correlation between anemia and negative clinical outcome, worse prognosis, and increased complications. The same conclusions may also be accepted for HRS. Anemia is not considered as one of the recognized factors for HRS. There is a difference between variceal bleeding and anemia. While the first causes acute volume depletion, anemia causes hypoxia. Treating anemia might be beneficial in improving renal functions in patients with HRS associated to other medical treatment modalities of HRS [5].

## 5. Conclusion

Renal dysfunction is a frequent complication in patients with end-stage chronic liver disease. The role of anemia in aggravating HRS in patients with cirrhosis is explained by hypoxia that can lead to microcirculatory renal ischemia. Other studies are required to determine if anemia is a precipitant factor for HRS or not.

## Figures and Tables

**Table 1:** Patients CTP groups.

	CTP-A	CTP-B	CTP-C
HRS-2 (*n*, %)	—	3 (33.3%)	6 (66.7%)
Non-HRS (*n*, %)	23 (56.1%)	10 (24.4%)	8 (19.5%)
Total (*n*, %)	23 (46%)	13 (26%)	14 (28%)

HRS-2, hepatorenal syndrome type 2; CTP, Child–Turcotte–Pugh.

**Table 2:** Hematologic findings.

	Total	HRS type 2	Non-HRS
Hemoglobin (g/dl)	10.24 ± 2.58	7.62 ± 2.45	10.82 ± 2.25
MCV (fl)	85.7 (70–113)	90.47 ± 12.06	84.72 ± 9.05
TCMH (pg)	28.04 ± 3.73	29.55 ± 3.71	27.71 ± 3.69
Hematocrit (%)	33.53 ± 7.23	29.22 ± 6.43	34.48 ± 7.12

HRS-2, hepatorenal syndrome type 2; MCV, mean corpuscular volume.

**Table 3:** Clinical and biological features of patients.

	Type 2 HRS	HRS	*p*
Age (median years)	68.33 ± 10.93	61.65 ± 11.9	0.129
Gender (M/F)	7/2	20/21	0.114
Ascites (present/total)	9/9	18/41	**0.002** ^*∗*^
Encephalopathy	5	3	**<0.001** ^*∗*^
Hemoglobin (g/dL)	7.62 ± 2.45	10.82 ± 2.25	**<0.001** ^*∗*^
Platelet (×10^3^/*μ*l)	147.88 ± 65.86	119.17 ± 64.14	0.232
Hematocrit (%)	29.22 ± 6.43	34.48 ± 7.12	**0.047** ^*∗*^
Creatinine (*μ*mol/l)	259.11 ± 151	73.65 ± 29.71	**0.003** ^*∗*^
Total bilirubin	87.11 ± 55.9	31.31 ± 34.34	**<0.001** ^*∗*^
Albumin (g/dL)	28.11 ± 2.42	32.77 ± 2.73	**<0.001** ^*∗*^
INR	1.64 ± 0.32	1.28 ± 0.22	**<0.001** ^*∗*^
CTP score	9.77 ± 1.3	6.95 ± 2.22	**0.001** ^*∗*^
MELD score	22.77 ± 4.43	11.5 ± 4.67	**<0.001** ^*∗*^

HRS, hepatorenal syndrome; M, male; F, female; INR, international normalized ratio; CTP, Child–Turcotte–Pugh; MELD, model of end-stage liver disease. ^*∗*^*p* ≤ 0.05.

**Table 4:** Logistic regression analysis.

	*A*	S.E	Wald	df	*p*	Exp (*B*)	95% CI for exp (*B*)	
							Lower	Upper
CTP	−0.764	0.574	1.770	1	0.183	0.466	0.151	1.436
MELD	0.608	0.264	5.300	1	**0.021** ^*∗*^	1.836	1.095	3.080
Hemoglobin	−0.946	0.480	3.884	1	**0.049** ^*∗*^	0.388	0.152	0.995
Constant	3.538	4.721	0.562	1	0.454	34.40		

CTP, Child–Turcotte–Pugh score; MELD, model of end-stage liver disease. ^*∗*^*p* ≤ 0.05.

**Table 5:** Clinical and biological features of survivor and nonsurvivor patients with type 2 HRS.

	HRS survivors	HRS nonsurvivors	*p*
Age (median years)	73.66 ± 10.11	65.66 ± 11.16	0.333
Gender (M/F)	3/0	4/2	0.257
Hemoglobin (g/dL)	9.9 ± 3.1	6.48 ± 1.36	**0.038** ^*∗*^
Hematocrit (%)	33.33 ± 1.52	27.16 ± 7.08	0.089
Creatinine (*μ*mol/l)	263.66 ± 159.15	256.83 ± 131.56	0.947
Total bilirubin	97.33 ± 77.46	82.00 ± 50.05	0.725
Albumin (g/dL)	28.11 ± 2.42	32.77 ± 2.73	0.098
CTP score	9.33 ± 1.52	10.00 ± 1.26	0.506
MELD score	21.00 ± 5.00	23.66 ± 4.32	0.432

HRS, hepatorenal syndrome; M, male; F, female; INR, international normalized ratio; CTP, Child–Turcotte–Pugh; MELD, model of end-stage liver disease. ^*∗*^*p* ≤ 0.05.

## Data Availability

Data used in this study are restricted.
